# Gut Microbiota Influences Meningioma Pathogenesis via Circulating Metabolites: A Two‐Sample Mendelian Randomization Study

**DOI:** 10.1002/brb3.70973

**Published:** 2025-10-15

**Authors:** Xuan Chen, Hui Tian, Lihui Han, Wenzhe Xu

**Affiliations:** ^1^ Department of Radiation Oncology Qilu Hospital of Shandong University Jinan China; ^2^ Department of Neurosurgery Qilu Hospital of Shandong University and Institute of Brain and Brain‐Inspired Science Shandong University Jinan China; ^3^ Shandong Key Laboratory of Brain Function Remodeling Jinan China

**Keywords:** gut microbiota, gut–brain axis, Mendelian randomization, meningioma, metabolites

## Abstract

**Background:**

Meningiomas are common solitary intracranial tumors without any apparent risk factors. In light of the growing interest in gut microbiome–brain tumor interactions, this investigation sought to explore potential links between intestinal microbial communities and meningioma pathogenesis, while also exploring the potential mediating role of specific metabolites.

**Methods:**

To investigate potential causal links between intestinal microbial communities and meningioma development, we implemented a bidirectional two‐sample Mendelian randomization (MR) approach examining 196 microbial taxa. Our analytical strategy incorporated a two‐stage MR methodology to pinpoint potential mediating factors. Furthermore, we performed comprehensive mediation analyses to assess the degree to which particular metabolic intermediates might influence the observed microbiota–meningioma associations.

**Results:**

Eight distinct microbial taxa exhibited potential causal associations with meningioma development. Among the identified taxa, genus *Lachnoclostridium* (odds ratio [OR]: 0.60; 95% confidence interval [CI]: 0.41, 0.89; *p* = 0.010) and class Lentisphaeria (OR: 0.73; 95% CI: 0.57, 0.95; *p* = 0.017) were suggestively associated with a reduced risk of meningioma, whereas family Oxalobacteraceae (OR: 1.28; 95% CI: 1.04, 1.58; *p* = 0.018) suggested a positive association with the risk of meningioma. An exploratory mediation analysis suggested that the relationships between genus *Lachnoclostridium*, class Lentisphaeria, and family Oxalobacteraceae and meningioma were mediated by the histidine to pyruvate ratio, hydroxymalonate, and 1‐linoleoylglycerol. Each of these accounted for 10.65%, 10.78%, and 11.82%, respectively.

**Conclusion:**

This investigation provides preliminary evidence that intestinal microbial communities play a contributory role in meningioma pathogenesis, with circulating metabolites potentially serving as key intermediaries in this microbiota–meningioma axis.

## Graphical abstract

Our Mendelian randomization study identifies protective (genus *Lachnoclostridium*, class Lentisphaeria) and risk‐associated (family Oxalobacteraceae) gut microbes causally linked to meningioma. These causal gut–brain relationships are mediated by specific metabolites (histidine to pyruvate ratio, hydroxymalonate, and 1‐linoleoylglycerol), revealing a novel mechanistic axis for meningioma pathogenesis.

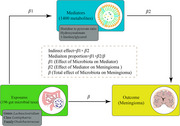

## 1 Introduction

Meningiomas constitute 41.7% of all primary intracranial tumors (Price et al. 2024), which may directly precipitate neurocognitive decline as a consequence of peritumoral edema and mass effect (Van Nieuwenhuizen et al. 2013; Zamanipoor Najafabadi et al. 2017). The excision of advanced‐stage tumors may prove challenging. Patients may experience considerable hospital costs and a markedly reduced quality of life (Jiang et al. 2022). Consequently, the discovery of reliable molecular markers and therapeutic targets holds substantial clinical value for enabling early detection and intervention for meningioma patients (L. Wang et al. 2020).

Current epidemiological evidence has identified several well‐documented risk factors associated with meningioma development. These include female sex, prior exposure to hormonal therapies, and a familial predisposition to the condition (Goh et al. 2024). Beyond the aforementioned genetic and external environmental factors, the gut–brain axis has been postulated as a pivotal mechanism through which gut microbiota may influence brain health (C. Liu et al. 2024). The gut microbiota modulates the function of host neurons through the production of various metabolites, including peptides, neurotransmitters, and neuroactive compounds (Dono et al. 2022). Dysbiosis of gut microbiome may result in the destruction of the gut barrier, leading to gut inflammation and a peripheral blood inflammatory response. This, in turn, may result in the destruction of the blood–brain barrier (BBB) function, causing a central inflammatory response and dysfunction of the hypothalamic–pituitary–adrenal axis, which may ultimately result in neurological dysfunction (Lv et al. 2022). Prior research has demonstrated distinct alterations in intestinal microbiome profiles when comparing meningioma patients to healthy controls (Jiang et al. 2022). Nevertheless, the potential causative role of gut microbial communities in meningioma pathogenesis has yet to be conclusively established.

Mendelian randomization (MR) employs the random assignment of single nucleotide polymorphisms (SNPs) during the process of conception to investigate the causal relationship between exposure factors and illness outcomes (Emdin et al. 2017). In this study, a two‐sample MR approach was implemented to systematically examine potential causal relationships between 196 distinct gut microbiota taxa and meningioma risk. Concurrently, a two‐step MR approach was employed to explore the potential role of significant mediators in this relationship. The preliminary findings of our study may facilitate the discovery of innovative treatment targets and contribute to a more profound comprehension of the etiology of meningioma. This, in turn, may prove beneficial in the context of prevention, recurrence, and the minimization of adverse effects through the intervention of gut microbiota and metabolites.

## 2 Methods

### 2.1 Study Design

**FIGURE 1:**
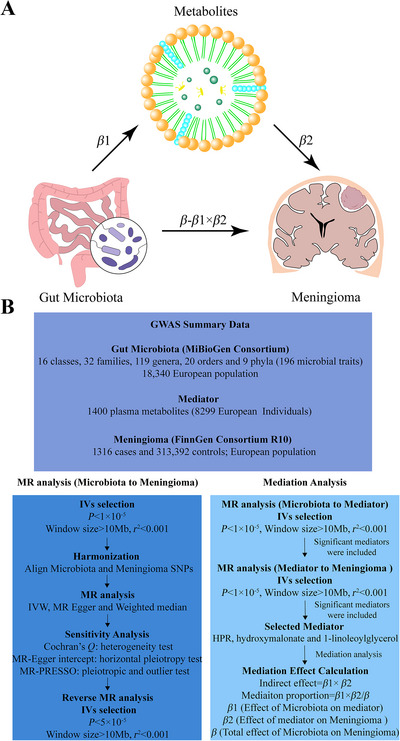
Exploring the relationship between gut microbiome and meningioma: a study based on the mediation analysis framework. (A) Schematic representation of the analytical framework employed in this investigation. The total effect (*β*) was partitioned into two components: (i) the indirect effect (*β*1 × *β*2), where *β*1 denotes the microbial influence on metabolite and *β*2 represents the metabolite's impact on meningioma, and (ii) direct effect (*β* − *β*1 × *β*2). The mediation proportion was quantified as the ratio of indirect to total effects. (B) Methodological workflow detailing the systematic approach for examining gut microbiome–meningioma relationships. The mediation framework specifically assessed the intermediary roles of HPR, hydroxymalonate, and 1‐LG in the observed microbial–meningioma association.

A two‐sample MR was conducted to examine the causal association between intestinal microbiota and meningioma. To ensure the validity and reliability of our findings, complementary analyses were performed, including reverse causation assessment and comprehensive sensitivity evaluations. In order to elucidate the intricate relationship between metabolic processes and intestinal microbial community, we systematically analyzed 1400 serum metabolites as potential mediators. This study utilized a two‐stage MR analytical framework, where mediation analysis was first conducted to identify key metabolic pathways. Subsequently, an effect decomposition model was employed to quantify the proportion of indirect effects mediated by gut microbiota metabolites in meningioma pathogenesis. The methodological design effectively delineated direct pathogenic pathways linking gut microbial communities to tumorigenesis while controlling for potential mediator interference. Figure 1 provides a visual representation of the study design and analytical steps employed in this investigation.

### 2.2 Data Source

Human gut microbiome genome‐wide association studies (GWAS) data were sourced from the MiBioGen consortium's open‐access repository. This study comprised 18,340 individuals of European ancestry and encompassed a total of 196 bacterial traits, including 9 phyla, 16 classes, 20 orders, 32 families, and 119 genera. Summary statistics for plasma metabolites were obtained from the GWAS Catalog (*n* = 8299) (Accession number: GCST90199621‐90201020). The FinnGen consortium R10 release data were utilized to obtain the meningioma statistics (1316 cases and 313,392 controls) (https://r10.finngen.fi/). To the best of our knowledge, the GWAS datasets for the exposure (gut microbiota), mediator (metabolites), and outcome (meningioma) were derived from separate cohorts, thereby precluding any sample overlap. Ethical approval was waived as this study analyzed publicly available GWAS summary data.

### 2.3 Instrumental Variable (IV) Selection Criteria

For each exposure variable, potential IVs were identified from GWAS data by selecting SNPs that achieved genome‐wide significance (*p* < 5 × 10^−8^). Due to limited IV availability for gut microbiota and metabolite analyses, we relaxed the significance threshold to *p* < 1 × 10^−5^—an accepted compromise in the field for microbiome GWAS data where conventional significance thresholds often yield insufficient IVs for powerful analysis, as demonstrated in recent methodological studies (X. Hu et al. 2024; P. Li et al. 2022). In the reverse MR analysis of meningioma, we adopted a threshold of *p* < 5 × 10^−5^ to ensure identification of at least 20 SNPs. We subsequently pruned SNPs to retain only those showing independence (window size > 10 Mb, *r* ^2^ < 0.001) based on European population linkage disequilibrium patterns. During data harmonization, we excluded palindromic SNPs and incompatible alleles.

To assess IV strength, we calculated *F*‐statistics using the formula: *F* = [*R* ^2^ (*n – k −* 1)]/[*k* (1 *− R* ^2^)], where *R* ^2^ denotes the proportion of exposure variance explained by the instrument, *n* represents sample size, and *k* indicates the number of SNPs. Following established criteria, *F*‐values exceeding 10 were considered indicative of sufficient instrument strength to minimize weak instrument bias (Staiger and Stock 1997).

### 2.4 MR Analysis

To examine potential causal associations between 196 microbial characteristics and meningioma risk, we performed bidirectional two‐sample MR analyses. Our analytical framework incorporated three complementary approaches: inverse variance weighted (IVW) as the primary method, supplemented by MR–Egger regression and weighted median estimation. The IVW method was prioritized due to its superior statistical power and more robust effect estimates under valid IV assumptions, consistent with established MR methodology (X. Chen et al. 2020; Xie et al. 2023; Larsson and Burgess 2022). Following initial analyses, we identified the most biologically relevant bacterial taxa for subsequent mediation investigation. To ensure methodological rigor, we conducted comprehensive sensitivity analyses and reverse MR assessments to evaluate potential horizontal pleiotropy and exclude reverse causation. For the reverse MR component, we implemented a *p* < 5.0 × 10^−5^ threshold for IV selection while maintaining identical analytical parameters to the forward MR framework. For multiple testing correction, the false discovery rate (FDR) correction, represented as *Q*‐value, was applied utilizing the Benjamini–Hochberg approach.

To identify the potential metabolites, we performed MR analyses to evaluate the causal associations between specific gut microbial taxa and a comprehensive panel of 1400 metabolites. Metabolites demonstrating significant associations were subsequently analyzed as exposures in secondary MR analyses with meningioma as the outcome. The most statistically significant metabolite was then selected for mediation analysis.

We implemented a two‐stage MR framework to examine the potential mediating role of plasma metabolites in the gut microbiota–meningioma association (Figure 1B). The IVW method was utilized to estimate: (1) the total effect of gut microbiota on meningioma risk (*β*), (2) the effect of gut microbiota on metabolite levels (*β*1), and (3) the effect of metabolites on meningioma risk (*β*2). Mediation effects were quantified using the product of coefficients method, where the indirect effect was calculated as *β*1 × *β*2. The direct effect was derived by subtracting the indirect effect from the total effect (*β* − *β*1 × *β*2). The proportion mediated was computed as the ratio of indirect to total effects (*β*1 × *β*2/*β*), as illustrated in Figure 1A.

### 2.5 Sensitivity Analysis

To evaluate heterogeneity among genetic instruments, Cochran's *Q* statistic was calculated using the IVW method (Yang et al. 2023). The presence of directional pleiotropy was examined through MR–Egger regression intercept analysis (Qi et al. 2023). Both leave‐one‐out SNP exclusion and the MR‐Pleiotropy Residual Sum and Outlier (MR‐PRESSO) approach were performed to identify and account for influential outlier variants that might affect causal estimates (L. Xu and Wang 2023). All statistical procedures were implemented in R software (version 4.2.1) utilizing the TwoSampleMR package (v0.5.7), with statistical significance threshold set at *p* < 0.05.

## 3 Results

### 3.1 Genetic Correlation Between Gut Microbiota and Meningioma

**FIGURE 2:**
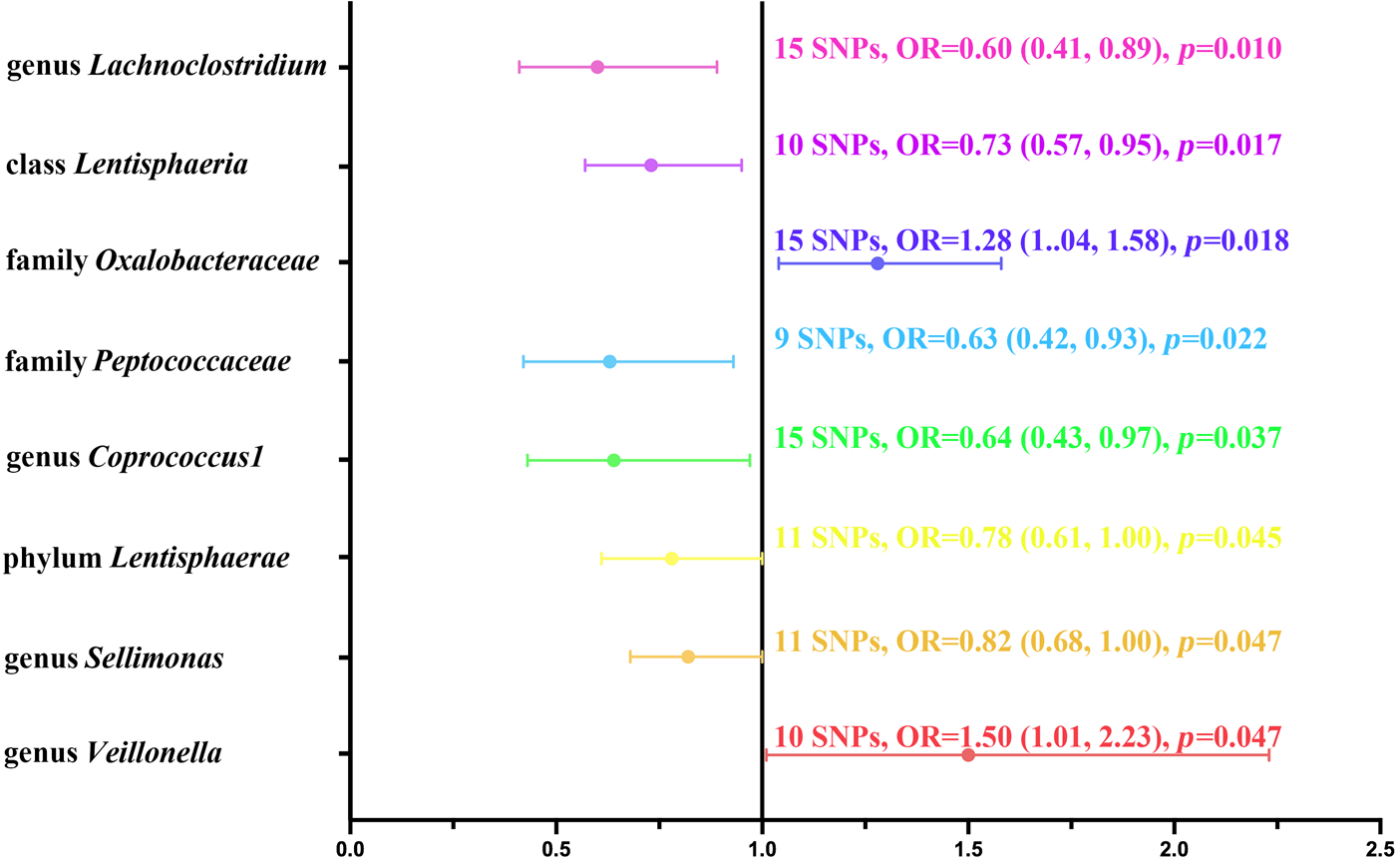
MR forest plot demonstrating causal associations between gut microbiome components and meningioma susceptibility.

To examine potential causal relationships between intestinal microbial communities and meningioma development, we conducted a bidirectional MR study analyzing 196 microbial taxa. Our initial analyses suggested statistically significant correlations between eight distinct bacterial characteristics and meningioma susceptibility (Figure 2).

Specifically, family Oxalobacteraceae (odds ratio [OR]: 1.28; 95% confidence interval [CI]: 1.04, 1.58; *p* = 0.018) and genus *Veillonella* (OR: 1.50; 95% CI: 1.01, 2.23; *p* = 0.047) suggested a potential positive association with meningioma. This indicates that an increased abundance of these taxa may potentially elevate the risk of meningioma. In contrast, genus *Lachnoclostridium* (OR: 0.60, 95% CI: 0.41, 0.89; *p* = 0.010), class Lentisphaeria (OR: 0.73; 95% CI: 0.57, 0.95; *p* = 0.017), family *Peptococcaceae* (OR: 0.63; 95% CI: 0.42, 0.93; *p* = 0.022), genus *Coprococcus1* (OR: 0.64, 95% CI: 0.43, 0.97; *p* = 0.037), phylum *Lentisphaerae* (OR: 0.78; 95% CI: 0.61, 1.00; *p* = 0.045), and genus *Sellimonas* (OR: 0.82; 95% CI: 0.68, 1.00; *p* = 0.047) were associated with a reduced risk of meningioma, hypothetically conferring protection against tumorigenesis. However, following FDR correction, no causal link between these genera and meningioma was established (Table S8). Cochran's *Q* test demonstrated the absence of instrument homogeneity (Table S1), while both MR–Egger regression (Table S2) and MR‐PRESSO analysis (Table S3) confirmed the absence of horizontal pleiotropy and outlier variants. Bidirectional MR analysis further established that meningioma status showed no causal influence on the eight examined gut microbiota taxa (Table S4). Although the causal associations for all eight microbial taxa did not survive multiple testing correction using FDR, we proceeded with a mediation analysis on the three taxa with the most suggestive uncorrected *p* values (genus *Lachnoclostridium*, class Lentisphaeria, and family Oxalobacteraceae) as an exploratory analysis to generate hypotheses for future research. This approach of investigating suggestive signals is a recognized strategy for hypothesis generation in genetic epidemiology (Zhu et al. 2018). Therefore, these results should be interpreted as preliminary and requiring independent validation in future studies.

### 3.2 Mediator Screening

In our investigation of potential mediators, we first examined the influence of intestinal microbial communities on a panel of 1400 metabolites. Our analysis revealed significant causal relationships between specific microbial taxa and numerous metabolites. Notably, genus *Lachnoclostridium* demonstrated associations with 93 distinct metabolites, while class Lentisphaeria and family Oxalobacteraceae showed connections with 52 and 64 metabolites, respectively (Tables S5–S7).

After identifying the influence of intestinal flora on metabolites, we further examined the potential mediation effects of these significant mediators on meningioma. With regard to the *Lachnoclostridium*‐associated metabolites, the results revealed a significant positive association between dimethyl sulfone and meningioma risk (OR: 1.30; 95% CI: 1.03, 1.65; *p* = 0.029). Conversely, an inverse relationship was observed for the histidine to pyruvate ratio (HPR) (OR = 0.76; 95% CI: 0.64–0.91; *p* = 0.002). While both mediators showed significance, HPR was included in further analysis due to its stronger statistical evidence (smaller *p* value) and because its effect direction was congruent with the total effect.

The class Lentisphaeria demonstrated a significant mediating influence on meningioma through two key metabolic intermediates: hydroxymalonate (OR: 1.32; 95% CI: 1.12, 1.57; *p* = 0.001) and 2,3‐dihydroxy‐2‐methylbutyrate (OR: 0.72; 95% CI: 0.52, 1.00; *p* = 0.049). However, the latter metabolite demonstrated substantial variability in effect estimates, as evidenced by the broad CIs and marginal statistical significance. Given these methodological limitations, we subsequently focused our investigation exclusively on hydroxymalonate for more robust analytical interpretation.

With regard to metabolites associated with the family Oxalobacteraceae, only 1‐linoleoylglycerol (1‐LG) (OR: 1.41; 95% CI: 1.03, 1.91; *p* = 0.030) exhibited an association with meningioma.

### 3.3 Mediation Analysis of Gut Microbiota on Meningioma

**FIGURE 3:**
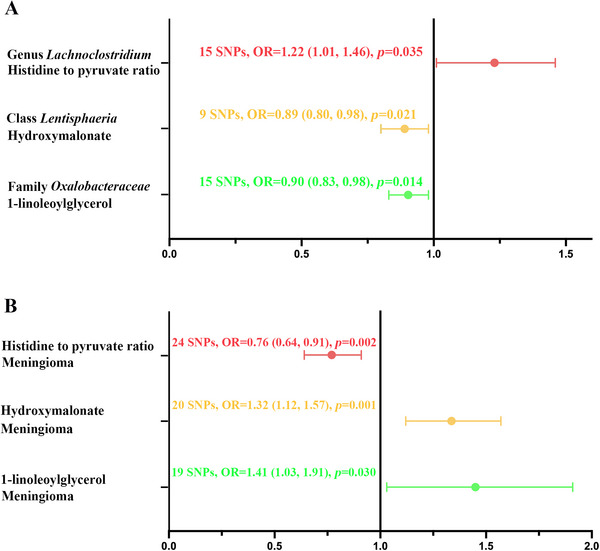
Mendelian randomization assessment: causal relationships between gut microbiota, intermediate biomarkers, and meningioma risk. (A) Forest plot demonstrating the causal associations between specific gut microbial taxa and potential mediating biomarkers. (B) Forest plot illustrating the causal effects of these mediating biomarkers on meningioma development.

Following comprehensive mediator screening, three pivotal metabolites were identified: HPR, hydroxymalonate, and 1‐LG (Figure 3).

Our analysis demonstrated a significant positive association between genus *Lachnoclostridium* and HPR (OR: 1.22; 95% CI: 1.01, 1.46; *p* = 0.035). Furthermore, HPR levels were inversely correlated with meningioma risk (OR: 0.76; 95% CI: 0.64, 0.91; *p* = 0.002).

The class Lentisphaeria was found to have a negative association with hydroxymalonate (OR: 0.89; 95% CI: 0.80, 0.98; *p* = 0.021), which in turn was significantly associated with meningioma (OR: 1.32; 95% CI: 1.12, 1.57; *p* = 0.001).

Our analysis revealed a significant association between family Oxalobacteraceae and elevated 1‐LG concentrations (OR: 1.09; 95% CI: 1.00, 1.19; *p* = 0.043). Furthermore, higher 1‐LG levels showed a statistically significant positive relationship with meningioma risk (OR: 1.41; 95% CI: 1.03, 1.91; *p* = 0.030). *F*‐statistics for the IVs across all exposures analyzed ranged from 17.04 to 41.91, satisfying the conventional strength threshold and reflecting instrument reliability (Table S9).

### 3.4 Mediation Proportion Between Gut Microbiota and Meningioma

Mediation analysis revealed gut microbiota–meningioma relationships through specific metabolites (Table 1). Notably, 10.7% of genus *Lachnoclostridium*’s meningioma association was mediated via HPR (total effect *β* = −0.507, direct effect [*β* − *β*1 × *β*2] = −0.453). Class Lentisphaeria showed 10.8% mediation through hydroxymalonate (total effect *β* = −0.311 vs. direct effect [*β* − *β*1 × *β*2] = −0.278). The family Oxalobacteraceae displayed 11.8% mediation via 1‐LG elevation (total effect *β* = 0.249, direct effect [*β* − *β*1 × *β*2] = 0.220). Each of these three metabolic mediators explained approximately 10%–12% of the total microbial–meningioma associations. This limited explanatory power suggested that these metabolites were likely partial mediators, and other unexplored biological pathways might also contribute to the observed associations.

**TABLE 1:** Mediation effect proportion.

Exposure	Mediator	Total effect (*β*)	Indirect effect (*β*1 × *β*2)	Direct effect (*β* − *β*1 × *β*2)	Proportion
Genus *Lachnoclostridium*	HPR	−0.507	−0.054	−0.453	10.65%
Class Lentisphaeria	Hydroxymalonate	−0.311	−0.034	−0.278	10.78%
Family Oxalobacteraceae	1‐LG	0.249	0.029	0.220	11.82%

Abbreviations: 1‐LG, 1‐linoleoylglycerol; HPR, histidine to pyruvate ratio.

## 4 Discussion

Emerging evidence suggests that dysbiosis of the intestinal microbiome may play a crucial etiological role in meningioma pathogenesis. This research employed two‐stage MR approach to elucidate the potential mediating effects of circulating metabolites in the association between gut microbiome composition and meningioma pathogenesis. The findings of the study suggested a protective association between genus *Lachnoclostridium* and meningioma risk. The results of the mediation analysis indicated that this connection was mediated by HPR. Furthermore, class Lentisphaeria has been suggested to potentially impede the progression of meningioma by reducing the hydroxymalonate level. Conversely, family Oxalobacteraceae may be associated with an increased risk of meningioma potentially via 1‐LG. The present study has identified novel bacterial and metabolic markers that may prove useful in the early detection of meningioma. Moreover, elucidating the exact mechanisms and intermediary pathways may provide critical perspectives for developing microbiota‐targeted therapeutic strategies against meningioma.

*Lachnoclostridium* is a recently delineated genus that belongs to the family Lachnospiraceae (J. Q. Liang et al. 2020; Sorbara et al. 2020), which is renowned for its capacity to produce short‐chain fatty acids (SCFAs). SCFAs represent key metabolic byproducts generated through microbial fermentation of dietary fiber within the intestinal lumen. They are often considered a key candidate mediator in neuroimmunoendocrine regulation (Dalile et al. 2019). SCFA can cross the BBB and influence central nervous system (CNS) functions, including microglial activation and inflammatory mediator synthesis (Ju et al. 2023). Previous study has identified a deficiency of SCFA‐producing probiotics in patients with meningioma, which is consistent with our findings. Furthermore, elevated pathogenic bacterial loads in meningioma patients demonstrated competitive inhibition against SCFA‐synthesizing microbiota, potentially destabilizing CNS immune homeostasis through microbial ecological remodeling (Jiang et al. 2022). *Lachnoclostridium* has been linked to Alzheimer's disease, insomnia, and moyamoya disease (Verhaar et al. 2021; Y. Li et al. 2023; X. Yu, Ge, et al. 2023). Nevertheless, research examining the relationship between this microbial taxon and meningioma remains limited. Our investigation provides innovative insights into the functional biology and underlying molecular mechanisms of this microbial species. The altered metabolites resulting from intestinal flora dysbiosis play a pivotal role in the pathogenesis of meningioma. The mediation analysis identified HPR as a vital mediator. Although *Lachnoclostridium* is a known SCFA producer, our analysis identified HPR rather than SCFAs as a significant mediator. This may be attributed to: (1) technical limitations in SCFA measurement in human samples, as SCFA levels are influenced by multiple factors including absorption and metabolic kinetics (Yamamura et al. 2020); (2) the complex, indirect mechanisms of SCFA action through immune regulation and epigenetic modifications rather than direct linear effects (Vinolo et al. 2011). Potential crosstalk between HPR and SCFA pathways may exist, as SCFAs can modulate host metabolism including amino acid balance (D. Zhang et al. 2023), possibly influencing HPR indirectly through microbial–host interactions. The potential role of histidine in the neoplastic process remains unclear. Previous studies have postulated that histidine could potentially facilitate the uptake of other amino acids. This transportation maintains amino acid pools at levels that mediate neoplasia (Koslinski et al. 2023). It is hypothesized that pyruvate carboxylase catalysis may promote the carbonylation of pyruvate into oxaloacetate, thereby providing tumor cells with augmented anaplerotic capacity and stimulating their anabolic metabolism. The acquisition of anaplerotic capacity by tumor cells is associated with accelerated growth and increased aggressiveness (Gondas et al. 2023). It remains unclear whether HPR's tumor‐suppressive effects are directly mediated through the proposed anaplerotic pathway. Therefore, future studies should focus on elucidating the signaling cascades underlying HPR's role in meningioma pathogenesis.

The findings of our study suggest a potential association between class Lentisphaeria and reduced risk of meningioma oncogenesis. This genus is responsible for the production of extracellular slime material and is classified within the phylum Lentisphaerae (Zhou et al. 2024). The class Lentisphaeria has been the subject of relatively little research attention. Nevertheless, the current research indicates that they are closely related to immunomodulation (Dong et al. 2024). This taxon has been demonstrated to exert disease‐mitigating properties on Parkinson's disease, coronary heart disease, and granulomatosis with polyangiitis (Ning et al. 2022; M. Xu et al. 2022; S. Chen et al. 2024). Additional investigations are warranted to clarify the underlying protective pathways. Our findings offer preliminary genetic evidence suggesting that certain serum metabolites mediated the protective effects of Lentisphaeria against meningioma. Mediation analysis revealed an inverse relationship between Lentisphaeria abundance and hydroxymalonate concentrations. Conversely, elevated serum levels of hydroxymalonate were linked with increased meningioma risk. The metabolite hydroxymalonate is known to have the potential to inhibit the efficacy of malic enzyme (ME), which plays a pivotal role in pyruvate metabolism (Z. Zhang, Qu, et al. 2024). Malate is oxidized by ME to generate pyruvate and CO_2_ (Du et al. 2022). This enzyme exerts crucial regulatory functions in cellular aging processes, lipogenesis, and the proliferation of numerous tumor cells (Z. Zhang, Yang, et al. 2024). In accordance with our findings, previous research has demonstrated that ME ablation facilitates colon carcinoma progression by suppression of CD8+ T lymphocyte‐mediated tumor surveillance (Z. Zhang, Yang, et al. 2024), further underscoring the tumor‐suppressive role of ME. However, findings from some studies are not entirely consistent. For example, emerging evidence indicates that mitochondrial biogenesis modulation by ME plays a crucial role in accelerating the pathogenesis of acute myeloid leukemia (Y. P. Wang et al. 2021). Furthermore, ME has been identified as a poor prognostic predictor for hepatocellular carcinoma (Du et al. 2022). These contradictory findings may be attributable to the context‐dependent roles of ME across distinct tissue types and the heterogeneous nature of tumors, where metabolic reprogramming pathways diverge significantly. In addition, the probable intricate interactions between the intestinal flora may elucidate the discrepancy between the genetically predicted outcomes and the clinical observations. In light of the pivotal role played by class Lentisphaeria in immunomodulation, further research is imperative to elucidate the impact of this taxon on the regulation of metabolic state and antitumor immunity.

Family Oxalobacteraceae serves as a crucial mediator in the metabolic breakdown of oxalate (Miller et al. 2019). Dysregulation of oxalate homeostasis has been associated with various pathological conditions, particularly renal disorders, inflammatory responses (both systemic and localized), as well as cerebrovascular and cardiovascular pathologies (Ermer et al. 2023). Our results align with prior studies suggesting a potential association between family Oxalobacteraceae and meningioma. Notably, this bacterial family appears to play a potential mediating role in reducing metformin's therapeutic efficacy against tumor growth (Z. Zhang, Wu, et al. 2024). Furthermore, family Oxalobacteraceae may exert neuroprotective effects, as evidenced by its inverse association with Parkinson's disease and delirium (Ning et al. 2022; H. Yu, Wan, et al. 2023). The precise molecular pathways through which Oxalobacteraceae influences CNS pathophysiology remain to be fully characterized and warrant further mechanistic exploration. Emerging evidence suggests that the pathogenesis of meningioma involves dysregulation of the gut–brain axis, mediated by microbial‐derived metabolites. Our results indicate that 1‐LG could potentially serve as an intermediary factor linking Oxalobacteraceae to meningioma. 1‐LG, a glycerol ester of linoleic acid, has been demonstrated to be a reproducible metabolite with the potential for inhibiting both drug‐resistant bacteria and cancer cell lines (Vu et al. 2018). This metabolite has the potential to treat pathological hemorrhage, ameliorate benign prostatic hyperplasia, and serve as a predictive marker for active tuberculosis (S. Li et al. 2019; H. Zhang et al. 2022; Xian et al. 2024). Furthermore, this bioactive metabolite also could work as a skin permeation enhancer (Kozaka et al. 2022). Nevertheless, the impact of 1‐LG on meningioma remains largely uninvestigated. A previous study demonstrated that a diet containing 1‐LG increased adrenaline secretion and energy expenditure by activating transient receptor potential vanilloid 1 (TRPV1) (Iwasaki et al. 2011). Meningioma specimens exhibited upregulated TRPV1 expression, mechanistically linked to tumor pathogenesis (Moutafidi et al. 2021). Furthermore, 1‐LG demonstrated favorable binding affinities with TP53, HSP90, JAK2, and caspase3, suggesting that this metabolite may influence meningioma progression by regulating multiple targets and pathways (M. M. Zhang et al. 2021). Further investigations utilizing randomized controlled trial designs will be essential for clarifying the fundamental biological pathways involved.

When interpreting the current findings, several methodological limitations warrant consideration. The taxonomic resolution of microbial exposure data was limited to the genus level, restricting our ability to explore causal relationships at finer taxonomic levels such as species or strain. Future microbiota GWAS could improve precision by employing advanced shotgun metagenomic sequencing. In addition, while the study cohort mainly consisted of individuals of European ancestry, potential residual population stratification effects may persist, making the generalizability of these results to non‐European populations uncertain and necessitating validation in diverse ethnic groups. The reverse MR analysis was further constrained by the modest sample size of gut microbiota data, highlighting the need for replication studies with larger cohorts to confirm potential reverse causation between meningioma and gut microbial composition.

The relatively modest sample size of meningioma cases in our investigation (*n* = 1316) potentially diminishes the statistical power and heightens the likelihood of encountering false‐negative results (Wu et al. 2024). In addition, the presence of weak instrument bias may be intensified under such circumstances (Arora et al. 2023), potentially distorting causal estimates in both forward and reverse MR analyses (Su et al. 2024). This limitation also extends to mediation MR, where insufficient sample size undermines the detection of intermediate pathways, as seen in integrated network pharmacology and expression quantitative trait loci (eQTL) studies (F. Wang et al. 2024). Future validation efforts should focus on collaborative initiatives that incorporate multi‐ancestry cohorts or utilize trans‐ethnic MR (TRMR) methodologies to improve statistical accuracy (Hou et al. 2025).

Notably, we used a relaxed significance threshold (*p* < 1 × 10^−5^) for IV selection to ensure an adequate number of IVs, given that genetic associations for certain microbial taxa are frequently limited—a challenge identified in MR investigations concerning complex traits (Böckerman et al. 2017). While this pragmatic compromise is widely accepted in the context of gut microbiome MR research (P. Li et al. 2022), it may inadvertently increase the risk of false‐positive results due to potential pleiotropic influences and the integration of weaker IVs (Y. Liang et al. 2023). Although this methodology diverges from the conventional genome‐wide significance threshold (*p* < 1 × 10^−8^), we conducted a thorough evaluation of the possible bias linked to the use of weak instruments by calculating *F*‐statistics for each IV, incorporating only those with an *F*‐value greater than 10, in alignment with established empirical standards (D. Liu et al. 2023). This empirical verification is consistent with methodological standards, as weak instruments (*F*‐value < 10) may lead to bias by failing to adequately account for confounding variables, while stronger instruments uphold the integrity of the estimations (Böckerman et al. 2017). Subsequent research should prioritize expanding cohort sizes to bolster both instrument strength and statistical power for more rigorous examinations of gut microbiota–meningioma associations, thereby advancing our understanding of gut–brain axis interactions.

The identified metabolites accounted for approximately 10.65%–11.82% of the total mediation effect, indicating that the majority of the effect may arise from alternative pathways. While our study identified potential mediating metabolites, this modest explanatory power underscores the complexity of microbiota–meningioma interactions. As established in cancer etiology research, microbial influences on cancer risk typically involve multiple interconnected mechanisms, including but not limited to metabolic hormone regulation, immune modulation, and chronic inflammation (Friedenreich et al. 2021). Our findings align with this paradigm by suggesting that the metabolites examined here are minor contributors within a broader network of biological pathways. This limitation arises from technical constraints of metabolomic platforms in detecting low‐abundance bioactive metabolites and the inherent combinatorial nature of metabolic networks, where single metabolites fail to represent systemic impacts (Y. Hu et al. 2022). Subsequent research endeavors ought to concentrate on previously unidentified microbial metabolites, SCFA‐mediated immune regulation, immunomodulatory signaling molecules, and microbiota‐derived neurotransmitters. This focus will aid in clarifying the involvement of gut–brain axis in meningioma pathogenesis (Dono et al. 2022; Dalile et al. 2019). In addition, investigations should consider other mediators, such as cytokines, sex hormones, and various metabolic pathways, to provide a more thorough understanding of the mechanistic foundations underlying these associations. Last but not least, we performed multiple testing corrections for the primary associations between gut microbiota and meningioma. However, these primary associations were no longer statistically significant following correction. Previous studies have suggested that this rigorous and conservative adjustment may overlook potential genera exhibiting a causal relationship with meningioma (Y. Liang et al. 2023). Nevertheless, we still accounted for the multiple testing results and subsequently reinterpreted the findings, specifically addressing this point in the manuscript's conclusions. Future research should expand the sample size to address these limitations.

## 5 Conclusion

Our analysis observed preliminary associations for eight microbial taxa with meningioma risk, suggesting potential risk‐enhancing and protective relationships. Specifically, the family Oxalobacteraceae and genus *Veillonella* were positively linked to higher meningioma incidence, implying a possible role in tumor development. In contrast, associations potentially indicative of protective effects were noted for genus *Lachnoclostridium*, class Lentisphaeria, family Peptococcaceae, *genus Coprococcus1*, phylum Lentisphaerae, and genus *Sellimonas*. Further mediation analysis identified specific metabolites—HPR, hydroxymalonate, and 1‐LG—as potential mediators in the relationships between certain microbial taxa (genus *Lachnoclostridium*, class Lentisphaeria, and family Oxalobacteraceae) and meningioma pathogenesis. This study provides initial insights into possible connections linking gut microbiota composition, circulating metabolites, and meningioma development, offering perspectives on how gut microbial communities might influence meningioma formation through metabolic pathways. These findings contribute to the mechanistic understanding of meningioma pathogenesis and highlight potential targets for future research and therapeutic strategies.

## Author Contributions

**Xuan Chen**: writing – original draft, data curation, formal analysis, funding acquisition. **Hui Tian**: writing – original draft, methodology, software, validation. **Lihui Han**: writing – review and editing, data curation, formal analysis, software, validation. **Wenzhe Xu**: writing – review and editing, funding acquisition, project administration, supervision.

## Conflicts of Interest

The authors declare no conflicts of interest.

## Peer Review

The peer review history for this article is available at https://publons.com/publon/10.1002/brb3.70973.

## Supporting information




**Supplementary Material**: brb370973‐sup‐0001‐SuppMatt.docx

## Data Availability

The datasets used in our study are available in FinnGen repository (https://r10.finngen.fi/), MiBioGen repository (https://mibiogen.gcc.rug.nl/), and GWAS Catalog (https://www.ebi.ac.uk/gwas/).
