# Supplementary material for: Gut Microbiota Influences Meningioma Pathogenesis via Circulating Metabolites: A Two‐Sample Mendelian Randomization Study
Source: Brain Behav. 2025 Oct 15;15(10):e70973. doi: 10.1002/brb3.70973 (PMC12528541; doi:10.1002/brb3.70973)
Supplement: Supplementary file 1 — Supplementary Material: brb370973‐sup‐0001‐SuppMatt.docx [file BRB3-15-e70973-s001.docx]

Supplementary Material

# Supplementary Figures and Tables

## Supplementary Table S1

**The heterogeneity of gut microbiota instrumental variables**

| **Bacterial taxa (exposure)** | **Cochran's Q** | **Df** | ***P*-value** |
| --- | --- | --- | --- |
| *genus Lachnoclostridium* | 9.42 | 13 | 0.741 |
| *class Lentisphaeria* | 7.42 | 8 | 0.492 |
| *family Oxalobacteraceae* | 11.37 | 13 | 0.580 |
| *family Peptococcaceae* | 7.73 | 7 | 0.357 |
| *genus Coprococcus1* | 15.25 | 12 | 0.228 |
| *phylum Lentisphaerae* | 9.65 | 9 | 0.379 |
| *genus Sellimonas* | 3.27 | 9 | 0.953 |
| *genus Veillonella* | 3.64 | 6 | 0.725 |

Df, degree of freedom

## Supplementary Table S2

**MR-PRESSO analysis for the 8 bacterial traits that have been identified to be associated with the risk of meningioma**

| **Bacterial taxa (exposure)** | **MR Analysis** | **Causal Estimate** | **SD** | **T** | ***P*-value** | **RSS_obs_** | **Global test *P*-value** | **Remove SNP** |
| --- | --- | --- | --- | --- | --- | --- | --- | --- |
| *genus Lachnoclostridium* | MR-PRESSO | -0.51 | 0.16 | -3.09 | 0.008 | 11.22 | 0.792 | None |
| *class Lentisphaeria* | MR-PRESSO | -0.311 | 0.12 | -2.62 | 0.028 | 9.03 | 0.646 | None |
| *family Oxalobacteraceae* | MR-PRESSO | 0.249 | 0.10 | 2.42 | 0.029 | 14.94 | 0.527 | None |
| *family Peptococcaceae* | MR-PRESSO | -0.465 | 0.20 | -2.30 | 0.051 | 12.30 | 0.333 | None |
| *genus Coprococcus1* | MR-PRESSO | -0.439 | 0.21 | -2.09 | 0.057 | 18.41 | 0.293 | None |
| *phylum Lentisphaerae* | MR-PRESSO | -0.247 | 0.12 | -2.02 | 0.071 | 11.67 | 0.501 | None |
| *genus Sellimonas* | MR-PRESSO | -0.20 | 0.058 | -3.44 | 0.006 | 4.06 | 0.972 | None |
| *genus Veillonella* | MR-PRESSO | 0.40 | 0.15 | 2.75 | 0.028 | 4.76 | 0.818 | None |

﻿MR, Mendelian randomization; MR-PRESSO analysis, MR Pleiotropy RESidual Sum and Outlier analysis; SD, standard deviation; RSS_obs_, observed residual sum of squares; SNP, single nucleotide polymorphism**﻿﻿**

## Supplementary Table S3

**Directional horizontal pleiotropy assessed by intercept term in MR Egger regression of the association between gut microbiota and meningioma**

| **Bacterial taxa (exposure)** | **Egger intercept** | **SE** | ***P*-value** |
| --- | --- | --- | --- |
| *genus Lachnoclostridium* | -0.02 | 0.04 | 0.609 |
| *class Lentisphaeria* | -0.01 | 0.07 | 0.899 |
| *family Oxalobacteraceae* | -0.08 | 0.06 | 0.191 |
| *family Peptococcaceae* | -0.06 | 0.05 | 0.264 |
| *genus Coprococcus1* | 0.02 | 0.04 | 0.540 |
| *phylum Lentisphaerae* | -0.02 | 0.07 | 0.740 |
| *genus Sellimonas* | 0.02 | 0.09 | 0.785 |
| *genus Veillonella* | -0.01 | 0.14 | 0.953 |

MR, Mendelian randomization; SE, standard error

## Supplementary Table S4

**Reverse MR estimates for the association between meningioma and gut microbiota**

| **Bacterial taxa (outcome)** | **Methods** | **No. of SNPs** | **OR** | **95% CI** | ***P*-value** |
| --- | --- | --- | --- | --- | --- |
| *genus Lachnoclostridium* | IVW | 34 | 0.99 | 0.97-1.01 | 0.242 |
| *class Lentisphaeria* | IVW | 32 | 1.01 | 0.96-1.06 | 0.805 |
| *family Oxalobacteraceae* | IVW | 42 | 0.99 | 0.96-1.03 | 0.656 |
| *family Peptococcaceae* | IVW | 42 | 0.99 | 0.97-1.02 | 0.505 |
| *genus Coprococcus1* | IVW | 44 | 1.01 | 0.99-1.03 | 0.154 |
| *phylum Lentisphaerae* | IVW | 42 | 1.02 | 0.98-1.07 | 0.361 |
| *genus Sellimonas* | IVW | 40 | 1.00 | 0.96-1.05 | 0.997 |
| *genus Veillonella* | IVW | 42 | 0.98 | 0.95-1.00 | 0.090 |

MR, Mendelian randomization; SNP, single nucleotide polymorphism; OR, odds ratio; CI, confidence interval; IVW, inverse variance weighted

## Supplementary Table S5

**Result of MR estimates for the association between** ***genus Lachnoclostridium* and metabolites**

| **Bacterial taxa (exposure)** | **Metabolites (outcome)** | ***P*-value** |
| --- | --- | --- |
| *genus Lachnoclostridium* | Adenosine 5'-monophosphate (AMP) levels | 0.017 |
| *genus Lachnoclostridium* | Phosphate levels (UKB data field 30810) | 0.016 |
| *genus Lachnoclostridium* | Adenosine 5'-diphosphate (ADP) to 5-oxoproline ratio | 0.029 |
| *genus Lachnoclostridium* | Adenosine 5'-monophosphate (AMP) to N-palmitoyl-sphingosine (d18:1 to 16:0) ratio | 0.044 |
| *genus Lachnoclostridium* | Adenosine 5'-monophosphate (AMP) to leucine ratio | 0.033 |
| *genus Lachnoclostridium* | Cerotoylcarnitine (C26) levels | 0.031 |
| *genus Lachnoclostridium* | Dihomo-linolenoylcarnitine (C20:3n3 or 6) levels | 0.010 |
| *genus Lachnoclostridium* | Dihomo-linoleoylcarnitine (C20:2) levels | 0.026 |
| *genus Lachnoclostridium* | Arachidonoylcarnitine (C20:4) levels | 0.012 |
| *genus Lachnoclostridium* | Spermidine levels | 0.004 |
| *genus Lachnoclostridium* | Alanine levels | 0.041 |
| *genus Lachnoclostridium* | Cytosine levels | 0.045 |
| *genus Lachnoclostridium* | Cis 3,4-methyleneheptanoate levels | 0.015 |
| *genus Lachnoclostridium* | Pipecolate levels | 0.037 |
| *genus Lachnoclostridium* | Sphinganine-1-phosphate levels | 0.009 |
| *genus Lachnoclostridium* | Androstenediol (3beta,17beta) disulfate (2) levels | 0.042 |
| *genus Lachnoclostridium* | 5alpha-androstan-3alpha,17beta-diol disulfate levels | 0.000 |
| *genus Lachnoclostridium* | 2s,3R-dihydroxybutyrate levels | 0.028 |
| *genus Lachnoclostridium* | Thioproline levels | 0.032 |
| *genus Lachnoclostridium* | X-25828 levels | 0.012 |
| *genus Lachnoclostridium* | X-25422 levels | 0.047 |
| *genus Lachnoclostridium* | 5alpha-androstan-3alpha,17beta-diol monosulfate (2) levels | 0.002 |
| *genus Lachnoclostridium* | Eugenol sulfate levels | 0.006 |
| *genus Lachnoclostridium* | Behenoyl sphingomyelin (d18:1/22:0) levels | 0.008 |
| *genus Lachnoclostridium* | Serine to alpha-ketobutyrate ratio | 0.008 |
| *genus Lachnoclostridium* | Threonine to alpha-ketobutyrate ratio | 0.024 |
| *genus Lachnoclostridium* | Histidine to alanine ratio | 0.004 |
| *genus Lachnoclostridium* | Glutamine to alanine ratio | 0.025 |
| *genus Lachnoclostridium* | Sphingosine 1-phosphate levels | 0.004 |
| *genus Lachnoclostridium* | Palmitoylcarnitine levels (Metabolon platform) | 0.040 |
| *genus Lachnoclostridium* | Homoarginine levels | 0.021 |
| *genus Lachnoclostridium* | Xanthurenate levels | 0.042 |
| *genus Lachnoclostridium* | Ribitol levels | 0.010 |
| *genus Lachnoclostridium* | Theobromine levels | 0.006 |
| *genus Lachnoclostridium* | Cis-4-decenoate (10:1n6) levels | 0.036 |
| *genus Lachnoclostridium* | Dimethyl sulfone levels | 0.024 |
| *genus Lachnoclostridium* | Carnitine C18:2 levels | 0.007 |
| *genus Lachnoclostridium* | X-21319 levels | 0.025 |
| *genus Lachnoclostridium* | X-18921 levels | 0.003 |
| *genus Lachnoclostridium* | 2'-o-methylcytidine levels | 0.004 |
| *genus Lachnoclostridium* | 2'-o-methyluridine levels | 0.032 |
| *genus Lachnoclostridium* | Phosphate to phosphoethanolamine ratio | 0.027 |
| *genus Lachnoclostridium* | Phosphate to glucose ratio | 0.010 |
| *genus Lachnoclostridium* | Phosphate to mannose ratio | 0.021 |
| *genus Lachnoclostridium* | Phosphate to fructose ratio | 0.040 |
| *genus Lachnoclostridium* | Phosphate to uridine ratio | 0.027 |
| *genus Lachnoclostridium* | Phosphate to alanine ratio | 0.004 |
| *genus Lachnoclostridium* | Adenosine 3',5'-cyclic monophosphate (cAMP) to adenosine 5'-monophosphate (AMP) ratio | 0.025 |
| *genus Lachnoclostridium* | Adenosine 5'-diphosphate (ADP) to phosphate ratio | 0.010 |
| *genus Lachnoclostridium* | 3-phosphoglycerate to phosphate ratio | 0.006 |
| *genus Lachnoclostridium* | Adenosine 5'-diphosphate (ADP) to arginine ratio | 0.021 |
| *genus Lachnoclostridium* | Adenosine 5'-monophosphate (AMP) to phenylalanine ratio | 0.012 |
| *genus Lachnoclostridium* | Adenosine 5'-monophosphate (AMP) to phosphate ratio | 0.002 |
| *genus Lachnoclostridium* | Histidine to trans-urocanate ratio | 0.014 |
| *genus Lachnoclostridium* | Glutarylcarnitine (c5-dc) levels | 0.048 |
| *genus Lachnoclostridium* | Oleoylcarnitine levels | 0.026 |
| *genus Lachnoclostridium* | X-14939 levels | 0.034 |
| *genus Lachnoclostridium* | X-13728 levels | 0.033 |
| *genus Lachnoclostridium* | X-26054 levels | 0.022 |
| *genus Lachnoclostridium* | 7-methylxanthine levels | 0.004 |
| *genus Lachnoclostridium* | 3,7-dimethylurate levels | 0.004 |
| *genus Lachnoclostridium* | X-11308 levels | 0.035 |
| *genus Lachnoclostridium* | Phosphate to oleoyl-linoleoyl-glycerol (18:1 to 18:2) [2] ratio | 0.025 |
| *genus Lachnoclostridium* | Alpha-ketoglutarate to pyruvate ratio | 0.014 |
| *genus Lachnoclostridium* | Adenosine 5'-monophosphate (AMP) to EDTA ratio | 0.033 |
| *genus Lachnoclostridium* | Adenosine 5'-diphosphate (ADP) to glutamine ratio | 0.026 |
| *genus Lachnoclostridium* | Adenosine 5'-monophosphate (AMP) to citrate ratio | 0.026 |
| *genus Lachnoclostridium* | Adenosine 5'-monophosphate (AMP) to glutamine ratio | 0.010 |
| *genus Lachnoclostridium* | Adenosine 5'-monophosphate (AMP) to threonine ratio | 0.007 |
| *genus Lachnoclostridium* | Adenosine 5'-monophosphate (AMP) to tryptophan ratio | 0.018 |
| *genus Lachnoclostridium* | Adenosine 5'-monophosphate (AMP) to glycine ratio | 0.033 |
| *genus Lachnoclostridium* | Adenosine 5'-monophosphate (AMP) to arginine ratio | 0.007 |
| *genus Lachnoclostridium* | Adenosine 5'-monophosphate (AMP) to tyrosine ratio | 0.019 |
| *genus Lachnoclostridium* | Adenosine 5'-monophosphate (AMP) to aspartate ratio | 0.048 |
| *genus Lachnoclostridium* | Adenosine 5'-monophosphate (AMP) to asparagine ratio | 0.022 |
| *genus Lachnoclostridium* | Adenosine 5'-monophosphate (AMP) to serine ratio | 0.007 |
| *genus Lachnoclostridium* | Adenosine 5'-monophosphate (AMP) to methionine ratio | 0.017 |
| *genus Lachnoclostridium* | Adenosine 5'-monophosphate (AMP) to histidine ratio | 0.006 |
| *genus Lachnoclostridium* | Alpha-ketoglutarate to alanine ratio | 0.026 |
| *genus Lachnoclostridium* | Adenosine 5'-monophosphate (AMP) to isoleucine ratio | 0.025 |
| *genus Lachnoclostridium* | Adenosine 5'-monophosphate (AMP) to valine ratio | 0.045 |
| *genus Lachnoclostridium* | Adenosine 5'-monophosphate (AMP) to cysteine ratio | 0.041 |
| *genus Lachnoclostridium* | Glutarate (C5-DC) to caprylate (8:0) ratio | 0.004 |
| *genus Lachnoclostridium* | Spermidine to (N(1) + N(8))-acetylspermidine ratio | 0.008 |
| *genus Lachnoclostridium* | Spermidine to N-acetylputrescine ratio | 0.028 |
| *genus Lachnoclostridium* | Spermidine to histidine ratio | 0.015 |
| *genus Lachnoclostridium* | Histidine to pyruvate ratio | 0.035 |
| *genus Lachnoclostridium* | Spermidine to phosphate ratio | 0.002 |
| *genus Lachnoclostridium* | Spermidine to carnitine ratio | 0.046 |
| *genus Lachnoclostridium* | Spermidine to ergothioneine ratio | 0.034 |
| *genus Lachnoclostridium* | Adenosine 5'-diphosphate (ADP) to glycine ratio | 0.039 |
| *genus Lachnoclostridium* | Phosphate to N-palmitoyl-sphingosine (d18:1 to 16:0) ratio | 0.023 |
| *genus Lachnoclostridium* | Adenosine 5'-diphosphate (ADP) to glycerol 3-phosphate ratio | 0.014 |

MR, Mendelian randomization

## Supplementary Table S6

**Result of MR estimates for the association between *class Lentisphaeria* and metabolites**

| **Bacterial taxa (exposure)** | **Metabolites (outcome)** | ***P*-value** |
| --- | --- | --- |
| *class Lentisphaeria* | Glutamine conjugate of C7H12O2 levels | 0.014 |
| *class Lentisphaeria* | Glycine conjugate of C10H14O2 (1) levels | 0.034 |
| *class Lentisphaeria* | Glycine conjugate of C10H12O2 levels | 0.002 |
| *class Lentisphaeria* | 2,3-dihydroxy-2-methylbutyrate levels | 0.026 |
| *class Lentisphaeria* | Hexanoylglutamine levels | 0.044 |
| *class Lentisphaeria* | 4-hydroxyphenylacetate levels | 0.019 |
| *class Lentisphaeria* | Phenylalanine levels | 0.050 |
| *class Lentisphaeria* | Uridine levels | 0.032 |
| *class Lentisphaeria* | X-23644 levels | 0.037 |
| *class Lentisphaeria* | Carotene diol (3) levels | 0.011 |
| *class Lentisphaeria* | Glycerate levels | 0.022 |
| *class Lentisphaeria* | Methyl indole-3-acetate levels | 0.015 |
| *class Lentisphaeria* | Cinnamoylglycine levels | 0.043 |
| *class Lentisphaeria* | X-18886 levels | 0.049 |
| *class Lentisphaeria* | Etiocholanolone glucuronide levels | 0.042 |
| *class Lentisphaeria* | Bilirubin (Z,Z) to etiocholanolone glucuronide ratio | 0.012 |
| *class Lentisphaeria* | Bilirubin (Z,Z) to glucuronate ratio | 0.011 |
| *class Lentisphaeria* | Histidine to asparagine ratio | 0.013 |
| *class Lentisphaeria* | 1-stearoyl-2-oleoyl-GPS (18:0/18:1) levels | 0.028 |
| *class Lentisphaeria* | 11beta-hydroxyandrosterone glucuronide levels | 0.019 |
| *class Lentisphaeria* | 11beta-hydroxyetiocholanolone glucuronide levels | 0.010 |
| *class Lentisphaeria* | Tartronate (hydroxymalonate) levels | 0.021 |
| *class Lentisphaeria* | Threonate levels | 0.032 |
| *class Lentisphaeria* | Maltotriose levels | 0.020 |
| *class Lentisphaeria* | Glucuronate levels | 0.047 |
| *class Lentisphaeria* | 4-hydroxy-2-oxoglutaric acid levels | 0.008 |
| *class Lentisphaeria* | Pseudouridine levels | 0.048 |
| *class Lentisphaeria* | Maltose levels in coronary artery disease | 0.021 |
| *class Lentisphaeria* | Alliin levels | 0.017 |
| *class Lentisphaeria* | 1-(1-enyl-palmitoyl)-2-arachidonoyl-GPE (p-16:0/20:4) levels | 0.016 |
| *class Lentisphaeria* | X-21312 levels | 0.030 |
| *class Lentisphaeria* | Phosphate to uridine ratio | 0.023 |
| *class Lentisphaeria* | Aspartate to asparagine ratio | 0.020 |
| *class Lentisphaeria* | Histidine to trans-urocanate ratio | 0.004 |
| *class Lentisphaeria* | Gulonate levels | 0.017 |
| *class Lentisphaeria* | 5,6-dihydrouridine levels | 0.013 |
| *class Lentisphaeria* | Phenylacetylglutamine levels | 0.019 |
| *class Lentisphaeria* | X-15486 levels | 0.011 |
| *class Lentisphaeria* | X-12830 levels | 0.021 |
| *class Lentisphaeria* | X-12822 levels | 0.006 |
| *class Lentisphaeria* | X-13729 levels | 0.044 |
| *class Lentisphaeria* | X-12844 levels | 0.019 |
| *class Lentisphaeria* | Alpha-tocopherol levels | 0.021 |
| *class Lentisphaeria* | Isovalerylcarnitine (C5) levels | 0.008 |
| *class Lentisphaeria* | X-11444 levels | 0.026 |
| *class Lentisphaeria* | Uridine to 2'-deoxyuridine ratio | 0.008 |
| *class Lentisphaeria* | Glucose to sucrose ratio | 0.028 |
| *class Lentisphaeria* | Glutamate to pyruvate ratio | 0.024 |
| *class Lentisphaeria* | Glutamate to alanine ratio | 0.031 |
| *class Lentisphaeria* | Trans-urocanate levels | 0.038 |
| *class Lentisphaeria* | Glutamate to cysteine ratio | 0.049 |
| *class Lentisphaeria* | Glutamine to asparagine ratio | 0.004 |

MR, Mendelian randomization

## Supplementary Table S7

**Result of MR estimates for the association between *family Oxalobacteraceae* and metabolites**

| **Bacterial taxa (exposure)** | **Metabolites (outcome)** | ***P*-value** |
| --- | --- | --- |
| *family Oxalobacteraceae* | 2-hydroxyarachidate levels | 0.040 |
| *family Oxalobacteraceae* | N-stearoyl-sphingadienine (d18:2/18:0) levels | 0.030 |
| *family Oxalobacteraceae* | N-palmitoyl-heptadecasphingosine (d17:1/16:0) levels | 0.033 |
| *family Oxalobacteraceae* | Ceramide (d18:1/17:0, d17:1/18:0) levels | 0.019 |
| *family Oxalobacteraceae* | Guanidinoacetate levels | 0.024 |
| *family Oxalobacteraceae* | Sphingosine levels | 0.003 |
| *family Oxalobacteraceae* | N-stearoyl-sphingosine (d18:1/18:0) levels | 0.027 |
| *family Oxalobacteraceae* | N-acetylputrescine levels | 0.024 |
| *family Oxalobacteraceae* | X-23659 levels | 0.036 |
| *family Oxalobacteraceae* | X-23739 levels | 0.031 |
| *family Oxalobacteraceae* | X-24556 levels | 0.030 |
| *family Oxalobacteraceae* | Ethyl alpha-glucopyranoside levels | 0.011 |
| *family Oxalobacteraceae* | Ximenoylcarnitine (C26:1) levels | 0.048 |
| *family Oxalobacteraceae* | N-acetyl-isoputreanine levels | 0.036 |
| *family Oxalobacteraceae* | 2-hydroxysebacate levels | 0.020 |
| *family Oxalobacteraceae* | 3-bromo-5-chloro-2,6-dihydroxybenzoic acid levels | 0.038 |
| *family Oxalobacteraceae* | Pentose acid levels | 0.039 |
| *family Oxalobacteraceae* | Bilirubin degradation product, C16H18N2O5 (2) levels | 0.044 |
| *family Oxalobacteraceae* | Allantoin levels | 0.000 |
| *family Oxalobacteraceae* | 5alpha-androstan-3alpha,17alpha-diol monosulfate levels | 0.003 |
| *family Oxalobacteraceae* | Cis-4-decenoylcarnitine (C10:1) levels | 0.036 |
| *family Oxalobacteraceae* | 1-(1-enyl-palmitoyl)-2-palmitoleoyl-GPC (P-16:0/16:1) levels | 0.032 |
| *family Oxalobacteraceae* | 1-(1-enyl-palmitoyl)-2-palmitoyl-GPC (P-16:0/16:0) levels | 0.030 |
| *family Oxalobacteraceae* | Arabitol/xylitol levels | 0.014 |
| *family Oxalobacteraceae* | Umbelliferone sulfate levels | 0.007 |
| *family Oxalobacteraceae* | 3-carboxy-4-methyl-5-propyl-2-furanpropanoate (cmpf) levels | 0.034 |
| *family Oxalobacteraceae* | N-acetylthreonine levels | 0.025 |
| *family Oxalobacteraceae* | Oxalate (ethanedioate) levels | 0.041 |
| *family Oxalobacteraceae* | 1-linoleoylglycerol (18:2) levels | 0.044 |
| *family Oxalobacteraceae* | Xanthurenate levels | 0.000 |
| *family Oxalobacteraceae* | N-acetylglutamate levels | 0.001 |
| *family Oxalobacteraceae* | Carboxyethyl-gaba levels | 0.011 |
| *family Oxalobacteraceae* | Blood sugar levels | 0.011 |
| *family Oxalobacteraceae* | 4-vinylphenol sulfate levels | 0.031 |
| *family Oxalobacteraceae* | Pyrraline levels | 0.028 |
| *family Oxalobacteraceae* | Dimethylarginine (sdma + adma) levels | 0.049 |
| *family Oxalobacteraceae* | 1-palmitoyl-2-docosahexaenoyl-gpc (16:0/22:6) levels | 0.015 |
| *family Oxalobacteraceae* | X-21796 levels | 0.025 |
| *family Oxalobacteraceae* | Phosphate to glucose ratio | 0.014 |
| *family Oxalobacteraceae* | Serine to alpha-tocopherol ratio | 0.042 |
| *family Oxalobacteraceae* | Adenosine 5'-monophosphate (AMP) to phosphate ratio | 0.047 |
| *family Oxalobacteraceae* | Cortisone to cortisol ratio | 0.037 |
| *family Oxalobacteraceae* | Sphingosine to phosphate ratio | 0.003 |
| *family Oxalobacteraceae* | Phenylacetylglutamine levels | 0.045 |
| *family Oxalobacteraceae* | X-12818 levels | 0.040 |
| *family Oxalobacteraceae* | X-13684 levels | 0.034 |
| *family Oxalobacteraceae* | X-16124 levels | 0.004 |
| *family Oxalobacteraceae* | Alpha-tocopherol levels | 0.037 |
| *family Oxalobacteraceae* | X-12007 levels | 0.005 |
| *family Oxalobacteraceae* | X-12740 levels | 0.042 |
| *family Oxalobacteraceae* | Tyrosine to pyruvate ratio | 0.027 |
| *family Oxalobacteraceae* | Alpha-ketoglutarate to pyruvate ratio | 0.021 |
| *family Oxalobacteraceae* | Phosphate to glutamine ratio | 0.017 |
| *family Oxalobacteraceae* | N-stearoyl-sphingosine (d18:1 to 18:0) to N-palmitoyl-sphinganine (d18:0 to 16:0) ratio | 0.014 |
| *family Oxalobacteraceae* | N-palmitoyl-sphingosine (d18:1 to 16:0) to N-stearoyl-sphingosine (d18:1 to 18:0) ratio | 0.014 |
| *family Oxalobacteraceae* | Cortisol to 4-cholesten-3-one ratio | 0.016 |
| *family Oxalobacteraceae* | 2,6-dihydroxybenzoic acid levels | 0.037 |
| *family Oxalobacteraceae* | 3-ethylcatechol sulfate (1) levels | 0.044 |
| *family Oxalobacteraceae* | Urea levels | 0.018 |
| *family Oxalobacteraceae* | Cysteine to alanine ratio | 0.040 |
| *family Oxalobacteraceae* | Glutamine to asparagine ratio | 0.020 |
| *family Oxalobacteraceae* | Spermidine to ergothioneine ratio | 0.039 |
| *family Oxalobacteraceae* | Carnitine to ergothioneine ratio | 0.042 |
| *family Oxalobacteraceae* | 3-phosphoglycerate to glycerate ratio | 0.028 |

MR, Mendelian randomization

## Supplementary Table S8

**P value of IVW analysis after FDR correction**

| **Exposure** | **Outcome** | ***P*-value** | **Q-value** |
| --- | --- | --- | --- |
| *genus Lachnoclostridium* | Meningioma | 0.010 | 1 |
| *class Lentisphaeria* | Meningioma | 0.017 | 1 |
| *family Oxalobacteraceae* | Meningioma | 0.018 | 1 |
| *family Peptococcaceae* | Meningioma | 0.022 | 1 |
| *genus Coprococcus1* | Meningioma | 0.037 | 1 |
| *phylum Lentisphaerae* | Meningioma | 0.045 | 1 |
| *genus Sellimonas* | Meningioma | 0.047 | 1 |
| *genus Veillonella* | Meningioma | 0.047 | 1 |

FDR, false discovery rate

## Supplementary Table S9

**F-statistics of Instrumental Variables for All Exposures**

| **Exposure** | **Outcome** | **SNP** | **F-statistics** |
| --- | --- | --- | --- |
| *genus Lachnoclostridium* | Meningioma | rs1031599 | 20.04 |
| *genus Lachnoclostridium* | Meningioma | rs12566975 | 19.58 |
| *genus Lachnoclostridium* | Meningioma | rs1528479 | 19.78 |
| *genus Lachnoclostridium* | Meningioma | rs1997204 | 19.94 |
| *genus Lachnoclostridium* | Meningioma | rs2385421 | 17.04 |
| *genus Lachnoclostridium* | Meningioma | rs3821998 | 20.14 |
| *genus Lachnoclostridium* | Meningioma | rs4738679 | 20.81 |
| *genus Lachnoclostridium* | Meningioma | rs6112314 | 26.96 |
| *genus Lachnoclostridium* | Meningioma | rs615997 | 23.09 |
| *genus Lachnoclostridium* | Meningioma | rs61915992 | 21.79 |
| *genus Lachnoclostridium* | Meningioma | rs62028349 | 19.67 |
| *genus Lachnoclostridium* | Meningioma | rs62285313 | 22.65 |
| *genus Lachnoclostridium* | Meningioma | rs72829893 | 19.20 |
| *genus Lachnoclostridium* | Meningioma | rs78068103 | 20.81 |
| *genus Lachnoclostridium* | Meningioma | rs789029 | 21.60 |
| *class Lentisphaeria* | Meningioma | rs1002941 | 20.22 |
| *class Lentisphaeria* | Meningioma | rs11770843 | 21.70 |
| *class Lentisphaeria* | Meningioma | rs17114848 | 22.06 |
| *class Lentisphaeria* | Meningioma | rs2031282 | 20.48 |
| *class Lentisphaeria* | Meningioma | rs2731834 | 21.33 |
| *class Lentisphaeria* | Meningioma | rs2825714 | 22.56 |
| *class Lentisphaeria* | Meningioma | rs62570196 | 24.18 |
| *class Lentisphaeria* | Meningioma | rs72640280 | 20.50 |
| *class Lentisphaeria* | Meningioma | rs73113483 | 20.65 |
| *class Lentisphaeria* | Meningioma | rs77599476 | 22.99 |
| *family Oxalobacteraceae* | Meningioma | rs111966731 | 20.94 |
| *family Oxalobacteraceae* | Meningioma | rs11246212 | 21.74 |
| *family Oxalobacteraceae* | Meningioma | rs12002250 | 19.40 |
| *family Oxalobacteraceae* | Meningioma | rs12509763 | 20.48 |
| *family Oxalobacteraceae* | Meningioma | rs1569853 | 24.70 |
| *family Oxalobacteraceae* | Meningioma | rs17138946 | 19.38 |
| *family Oxalobacteraceae* | Meningioma | rs36057338 | 20.73 |
| *family Oxalobacteraceae* | Meningioma | rs4428215 | 29.80 |
| *family Oxalobacteraceae* | Meningioma | rs561239 | 19.86 |
| *family Oxalobacteraceae* | Meningioma | rs6000536 | 24.05 |
| *family Oxalobacteraceae* | Meningioma | rs62435498 | 20.52 |
| *family Oxalobacteraceae* | Meningioma | rs736744 | 27.71 |
| *family Oxalobacteraceae* | Meningioma | rs7993559 | 20.97 |
| *family Oxalobacteraceae* | Meningioma | rs80330081 | 19.59 |
| *family Oxalobacteraceae* | Meningioma | rs934049 | 21.14 |
| *genus Lachnoclostridium* | histidine-to-pyruvate ratio | rs1031599 | 20.04 |
| *genus Lachnoclostridium* | histidine-to-pyruvate ratio | rs12566975 | 19.58 |
| *genus Lachnoclostridium* | histidine-to-pyruvate ratio | rs1528479 | 19.78 |
| *genus Lachnoclostridium* | histidine-to-pyruvate ratio | rs1997204 | 19.94 |
| *genus Lachnoclostridium* | histidine-to-pyruvate ratio | rs2385421 | 17.04 |
| *genus Lachnoclostridium* | histidine-to-pyruvate ratio | rs3821998 | 20.14 |
| *genus Lachnoclostridium* | histidine-to-pyruvate ratio | rs4738679 | 20.81 |
| *genus Lachnoclostridium* | histidine-to-pyruvate ratio | rs6112314 | 26.96 |
| *genus Lachnoclostridium* | histidine-to-pyruvate ratio | rs615997 | 23.09 |
| *genus Lachnoclostridium* | histidine-to-pyruvate ratio | rs61915992 | 21.79 |
| *genus Lachnoclostridium* | histidine-to-pyruvate ratio | rs62028349 | 19.67 |
| *genus Lachnoclostridium* | histidine-to-pyruvate ratio | rs62285313 | 22.65 |
| *genus Lachnoclostridium* | histidine-to-pyruvate ratio | rs72829893 | 19.20 |
| *genus Lachnoclostridium* | histidine-to-pyruvate ratio | rs78068103 | 20.81 |
| *genus Lachnoclostridium* | histidine-to-pyruvate ratio | rs789029 | 21.60 |
| *class Lentisphaeria* | hydroxymalonate | rs1002941 | 20.23 |
| *class Lentisphaeria* | hydroxymalonate | rs11770843 | 21.70 |
| *class Lentisphaeria* | hydroxymalonate | rs17114848 | 22.07 |
| *class Lentisphaeria* | hydroxymalonate | rs2731834 | 21.33 |
| *class Lentisphaeria* | hydroxymalonate | rs2825714 | 22.57 |
| *class Lentisphaeria* | hydroxymalonate | rs62570196 | 24.19 |
| *class Lentisphaeria* | hydroxymalonate | rs72640280 | 20.51 |
| *class Lentisphaeria* | hydroxymalonate | rs73113483 | 20.65 |
| *class Lentisphaeria* | hydroxymalonate | rs77599476 | 23.00 |
| *family Oxalobacteraceae* | 1-linoleoylglycerol | rs111966731 | 20.95 |
| *family Oxalobacteraceae* | 1-linoleoylglycerol | rs11246212 | 21.74 |
| *family Oxalobacteraceae* | 1-linoleoylglycerol | rs12002250 | 19.41 |
| *family Oxalobacteraceae* | 1-linoleoylglycerol | rs12509763 | 20.49 |
| *family Oxalobacteraceae* | 1-linoleoylglycerol | rs1569853 | 24.71 |
| *family Oxalobacteraceae* | 1-linoleoylglycerol | rs17138946 | 19.39 |
| *family Oxalobacteraceae* | 1-linoleoylglycerol | rs36057338 | 20.74 |
| *family Oxalobacteraceae* | 1-linoleoylglycerol | rs4428215 | 29.81 |
| *family Oxalobacteraceae* | 1-linoleoylglycerol | rs561239 | 19.86 |
| *family Oxalobacteraceae* | 1-linoleoylglycerol | rs6000536 | 24.06 |
| *family Oxalobacteraceae* | 1-linoleoylglycerol | rs62435498 | 20.53 |
| *family Oxalobacteraceae* | 1-linoleoylglycerol | rs736744 | 27.72 |
| *family Oxalobacteraceae* | 1-linoleoylglycerol | rs7993559 | 20.97 |
| *family Oxalobacteraceae* | 1-linoleoylglycerol | rs80330081 | 19.59 |
| *family Oxalobacteraceae* | 1-linoleoylglycerol | rs934049 | 21.15 |
| histidine-to-pyruvate ratio | Meningioma | rs10931943 | 19.79 |
| histidine-to-pyruvate ratio | Meningioma | rs1260326 | 39.10 |
| histidine-to-pyruvate ratio | Meningioma | rs13001387 | 20.95 |
| histidine-to-pyruvate ratio | Meningioma | rs139522408 | 27.39 |
| histidine-to-pyruvate ratio | Meningioma | rs140850460 | 22.62 |
| histidine-to-pyruvate ratio | Meningioma | rs143350489 | 21.21 |
| histidine-to-pyruvate ratio | Meningioma | rs185423687 | 20.33 |
| histidine-to-pyruvate ratio | Meningioma | rs2502924 | 21.81 |
| histidine-to-pyruvate ratio | Meningioma | rs4905781 | 20.96 |
| histidine-to-pyruvate ratio | Meningioma | rs55712631 | 21.36 |
| histidine-to-pyruvate ratio | Meningioma | rs56024660 | 21.10 |
| histidine-to-pyruvate ratio | Meningioma | rs57538453 | 22.23 |
| histidine-to-pyruvate ratio | Meningioma | rs62175693 | 21.87 |
| histidine-to-pyruvate ratio | Meningioma | rs6501099 | 19.74 |
| histidine-to-pyruvate ratio | Meningioma | rs6742589 | 23.44 |
| histidine-to-pyruvate ratio | Meningioma | rs72790279 | 21.96 |
| histidine-to-pyruvate ratio | Meningioma | rs73022569 | 19.82 |
| histidine-to-pyruvate ratio | Meningioma | rs74970748 | 20.06 |
| histidine-to-pyruvate ratio | Meningioma | rs7648958 | 23.47 |
| histidine-to-pyruvate ratio | Meningioma | rs77170467 | 19.89 |
| histidine-to-pyruvate ratio | Meningioma | rs77525865 | 20.63 |
| histidine-to-pyruvate ratio | Meningioma | rs79585763 | 21.09 |
| histidine-to-pyruvate ratio | Meningioma | rs79888339 | 20.03 |
| histidine-to-pyruvate ratio | Meningioma | rs9792562 | 30.32 |
| hydroxymalonate | Meningioma | rs114537403 | 19.74 |
| hydroxymalonate | Meningioma | rs11691183 | 22.15 |
| hydroxymalonate | Meningioma | rs117550283 | 19.63 |
| hydroxymalonate | Meningioma | rs11766962 | 23.69 |
| hydroxymalonate | Meningioma | rs140591764 | 23.34 |
| hydroxymalonate | Meningioma | rs145469088 | 25.01 |
| hydroxymalonate | Meningioma | rs151096450 | 20.08 |
| hydroxymalonate | Meningioma | rs17688758 | 21.36 |
| hydroxymalonate | Meningioma | rs191302291 | 24.35 |
| hydroxymalonate | Meningioma | rs191390190 | 20.15 |
| hydroxymalonate | Meningioma | rs276286 | 23.15 |
| hydroxymalonate | Meningioma | rs55703838 | 20.47 |
| hydroxymalonate | Meningioma | rs5996848 | 21.62 |
| hydroxymalonate | Meningioma | rs62381620 | 41.91 |
| hydroxymalonate | Meningioma | rs72801867 | 21.18 |
| hydroxymalonate | Meningioma | rs73396929 | 23.02 |
| hydroxymalonate | Meningioma | rs7478 | 19.77 |
| hydroxymalonate | Meningioma | rs756874 | 21.79 |
| hydroxymalonate | Meningioma | rs77181656 | 19.67 |
| hydroxymalonate | Meningioma | rs9599028 | 21.75 |
| 1-linoleoylglycerol | Meningioma | rs10006032 | 20.67 |
| 1-linoleoylglycerol | Meningioma | rs10072491 | 20.88 |
| 1-linoleoylglycerol | Meningioma | rs10735636 | 19.56 |
| 1-linoleoylglycerol | Meningioma | rs11033752 | 19.59 |
| 1-linoleoylglycerol | Meningioma | rs11160050 | 20.18 |
| 1-linoleoylglycerol | Meningioma | rs113627835 | 20.69 |
| 1-linoleoylglycerol | Meningioma | rs114473062 | 21.20 |
| 1-linoleoylglycerol | Meningioma | rs117168942 | 19.65 |
| 1-linoleoylglycerol | Meningioma | rs12447287 | 20.51 |
| 1-linoleoylglycerol | Meningioma | rs12858688 | 20.28 |
| 1-linoleoylglycerol | Meningioma | rs17410962 | 23.41 |
| 1-linoleoylglycerol | Meningioma | rs2943179 | 19.71 |
| 1-linoleoylglycerol | Meningioma | rs56354314 | 20.54 |
| 1-linoleoylglycerol | Meningioma | rs62385039 | 21.82 |
| 1-linoleoylglycerol | Meningioma | rs6475038 | 20.48 |
| 1-linoleoylglycerol | Meningioma | rs75445316 | 23.76 |
| 1-linoleoylglycerol | Meningioma | rs77982886 | 24.87 |
| 1-linoleoylglycerol | Meningioma | rs964184 | 25.88 |
| 1-linoleoylglycerol | Meningioma | rs9894638 | 19.87 |

## Supplementary Figure S1


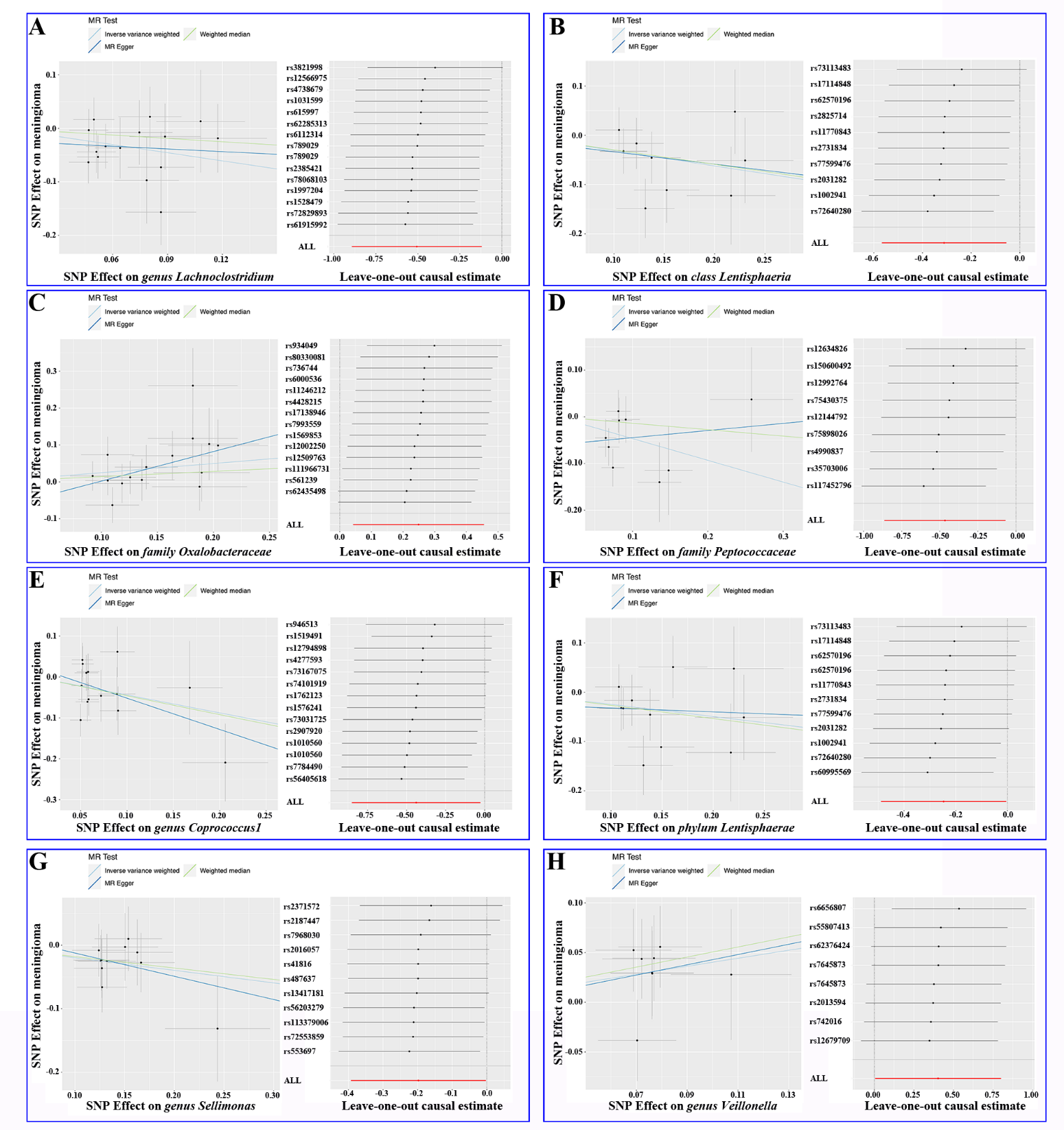


**Supplementary Figure S1.** Scatter plot and leave-one-out plot for the causal association between (A) *genus Lachnoclostridium*, (B) *class Lentisphaeria*, (C) *family Oxalobacteraceae*, (D) *family Peptococcaceae,* (E) *genus Coprococcus1,* (F) *phylum Lentisphaerae*, (G) *genus Sellimonas*, (H) *genus Veillonella* and meningioma.
